# Usefulness of the DETECT program for assessing the internal structure of dimensionality in simulated data and results of the Korean nursing licensing examination

**DOI:** 10.3352/jeehp.2017.14.32

**Published:** 2017-12-27

**Authors:** Dong Gi Seo, Younyoung Choi, Sun Huh

**Affiliations:** 1 Department of Psychology, College of Social Science, Hallym University, Chuncheon, Korea; 2 Department of Psychology, Hanyang Cyber University, Seoul, Korea; 3 Department of Parasitology and Institute of Medical Education, College of Medicine, Hallym University, Chuncheon, Korea; The Catholic University of Korea, Seoul, Korea

**Keywords:** Dimensionality, Korea, Licensure, Nursing, Simulation

## Abstract

**Purpose:**

The dimensionality of examinations provides empirical evidence of the internal test structure underlying the responses to a set of items. In turn, the internal structure is an important piece of evidence of the validity of an examination. Thus, the aim of this study was to investigate the performance of the DETECT program and to use it to examine the internal structure of the Korean nursing licensing examination.

**Methods:**

Non-parametric methods of dimensional testing, such as the DETECT program, have been proposed as ways of overcoming the limitations of traditional parametric methods. A non-parametric method (the DETECT program) was investigated using simulation data under several conditions and applied to the Korean nursing licensing examination.

**Results:**

The DETECT program performed well in terms of determining the number of underlying dimensions under several different conditions in the simulated data. Further, the DETECT program correctly revealed the internal structure of the Korean nursing licensing examination, meaning that it detected the proper number of dimensions and appropriately clustered the items within each dimension.

**Conclusion:**

The DETECT program performed well in detecting the number of dimensions and in assigning items for each dimension. This result implies that the DETECT method can be useful for examining the internal structure of assessments, such as licensing examinations, that possess relatively many domains and content areas.

## Introduction

An important aspect of test development is investigating the underlying structure of the test. The resulting information provides evidence about whether test items measure the construct(s) for which they were designed. This concept has traditionally been referred to as construct validity. Collecting evidence related to the internal structure of a test is likewise an important part of validation. For this purpose, factor analysis methods have been proposed as a powerful tool for exploring a test’s underlying structure. Additionally, in recent years, item response theory (IRT) has been applied in a broad substantive context, such as achievement, psychiatric, and medical license/certification examinations. Even though IRT has many advantages in terms of measurement, it requires the following strong assumptions: unidimensionality and the local independence assumption. More technically, unidimensionality is satisfied when item responses are independent after controlling for a single latent variable [1]. Indeed, the validity of IRT applications (linking, model-fit, parameter estimation, scoring, and adaptive testing) depends to a considerable extent on the unidimensionality assumption. Therefore, extensive research has investigated whether the assumptions of IRT hold in real test situations [2]. Studies have suggested that if the assumptions underlying IRT are questionable, it would be suitable to build a multidimensional IRT model to measure the underlying traits. Consequently, the dimensionality of the test structure must be examined before applying an IRT model. Furthermore, since an assessment of dimensionality evaluates test validity—that is, whether what we measure is what we want to measure—dimensionality testing of educational and psychological tests is essential.

Many programs have been proposed to examine the dimensionality of an assessment based on the procedures and concepts of cluster recovery. That is, if items measure a similar construct, they will be clustered, and each clustered item set can be interpreted as a dimension [3]. In general, these approaches can be categorized into partialinformation and full-information approaches from the perspective of factor analysis, while the DETECT program utilizes a non-parametric approach. Although the DETECT program has been proposed, its performance has not been fully investigated in various testing conditions. Therefore, in this study, the performance of the nonparametric DETECT program was examined using simulated and real data. First, a simulation study was conducted to investigate the performance of the DETECT program in different testing conditions. Second, an empirical study was conducted using data collected from the Korean nursing licensing examination.

## Methods

### Study design

This study involved the analysis of simulated data and real data with a unidimensionality test program.

### Simulated data

Data were simulated to follow the compensatory multidimensional 2-parameter logistic model and the 3-parameter logistic model as follows [4]:

(1) Pxij =1aiβiθj=expa'iθj+βi1+expa'iθj+βi

where *x_ij_* is the score (0,1) on item *i* by person *j*,

*a_i_* is the vector of item discrimination parameters,

*β_i_*, is a scalar parameter (intercept) that is related to the difficulty of the item,

*θ_j_* is the vector of ability parameters for person *j*.

The item discrimination parameters were simulated with log-normal distributions (mean= 0, standard deviation= 0.25). The guessing parameter (*c*-parameter) was set to 0.25 for the 3-parameter logistic IRT models and set to zero for the 2-parameter logistic IRT models. For the 2-dimensional models, discrimination values were calculated from the multidimensional discrimination (MDISC) values such that the first 10 items had an angle of 15° with the first factor axis and 75° with the second factor axis; items 11–20 had angles of 30° and 60° with the first and second factors, respectively; items 21–30 had angles of 60° and 30° with the first and second factors, respectively; and items 31–40 had angles of 75° and 15° with the first and second factors, respectively. With this design, each factor had some items that measured it more than the other factors. For the 4-dimensional models, each item was assigned to load on only 2 factors. The first 10 items were assigned to load primarily on the first factor and secondarily on the second factor, the second 10 items primarily on the second and secondarily on the third factor, the third 10 items primarily on the third and secondarily on the fourth factor, and the last 10 items primarily on the fourth and secondarily on the first factor with angles of 15° and 75°, respectively. Difficulty values were generated as the product of the MDISC and a random draw from a standard normal distribution. One hundred test forms were replicated for each condition, with the same number of items (40) on each test.

Different dimensionality assessments might have different degrees of capability to detect dimensions depending on the correlation values among dimensions in a given test. Two conditions were generated to explore different levels of multidimensionality in a test. Strong multidimensionality was indicated by applying an average correlation of 0.3, while mild multidimensionality was indicated by applying an average correlation of 0.7.

A traditional non-linear factor analysis assumes a multivariate normal distribution. As the number of examines increases, the distribution of theta will approach a normal distribution by the central limit theorem. Therefore, traditional methods can be sensitive to different numbers of examines. For this reason, this study considered 2 numbers of examinees (100 and 1,000 examinees). All responses according to the above conditions were generated by R (http://www.Rproject.org) [5] following methods described in previous data generation research [7,8].

### Real data

A study of real data was conducted using data collected from the Korean nursing licensing examination administered in January 2014. This examination contained 8 different subjects with different item numbers (Table 1). Each subject contains unique items that candidates are required to learn as content objectives. Table 1 shows the domain specification, including the number of items. The number of examinees of the real data was 16,085 and its number of items was 330. The real data are available in Supplement 1.

### Technical information

The DETECT program was provided by Measured Progress (https://psychometrics.onlinehelp.measuredprogress.org/research) [9,10] and an exploratory DETECT analysis can be conducted using the expl.detect function in “sirt” packages [6] in R program [5]. It examines the extent of the multidimensional simple structure of an assessment. The DETECT program relies on the covariance of items, conditional on an estimate of the unidimensional ability measured by an assessment. It has 2 indices for dimensionality: Dp* and *r*. A maximum DETECT value (Dp*) less than 0.1 indicates essential unidimensionality, while values greater than 1.0 indicate sizable multidimensionality [2]. Values of *r* greater than 0.8 indicate an approximately simple structure of multidimensionality.

### Statistics

The DETECT program examines the extent of the multidimensional simple structure of an assessment [9,10]. The DETECT program relies on the covariance of items, conditioned on an estimate of the unidimensional ability *θ_a_* for a given composite direction α to be measured by the examination. In 2 dimensions, the DETECT program indicates that items in the same cluster based on *θ_a_* have positive conditional covariance, while items in different clusters based on *θ_a_* have negative conditional covariance. The DETECT program then portions items in a way that maximizes the number of positively covarying items placed in the same clusters and the number of negatively covarying items placed in different clusters. The theoretical DETECT index of dimensionality for a given composite *θ_a_* is defined as:

(2) Da(p)2n(n-1)ΣδijECovXiXjθa

where P is any partition of the test;

δijp=1 if Xi,Xj are the same cluster P -1 = otherwise CovXi,Xjθa=the conditional covariance of Xi, Xj given θa.

When *θ_a_* is the test composite *θ_a_*, Da(P) is the theoretical DETECT index evaluated at partition P. The partition that maximizes Da(P) is denoted as P*.

### Ethical approval

The requirement to obtain informed consent was exempted by the Institutional Review Board of Hallym University (HIRB-2015-047). There were no person-identifiable data.

## Results

### Results of the simulation study

Specific patterns were found in the performance of the DETECT program. First, all Dp* values were greater than 0.1, indicating that all conditions possessed multidimensionality. Second, when the response data contained the guessing parameter, the Dp* values tended to be higher than without the guessing parameter. Third, the higher the correlation level was, the lower the Dp* values tended to be. Fourth, when the number of dimensions was larger, the Dp* values tended to be higher. Table 2 presents various Dp* values depending on whether the guessing parameter was included or absent. In general, Dp* was higher when the guessing parameter existed. The condition of 1,000 examinees with a 0.7 correlation level and 2 dimensions and the condition of 1,000 examinees with a 0.7 correlation level and 4 dimensions had different patterns than the other conditions. When the correlations among dimensions were higher—that is, when the associations among dimensions were strong—DETECT tended to determine an inaccurate number of dimensions. That is, the DETECT procedure was less sensitive for detecting the number of dimensions than it was for the effects of the guessing parameter and correlation among dimensions. Table 2 shows the performance of the DETECT program in terms of the number of dimensions, correlation levels among dimensions, sample size, and the presence or absence of the guessing parameter. The DETECT program performed relatively well under various conditions. If the number of examinees is large and the correlations among dimensions are relatively weak, the DETECT procedure tended to determine the number of dimensions well. However, the DETECT program tended to overestimate the number of dimensions when the guessing parameter was included.

### Results of the real data study

The real data study was conducted using results of the Korean nursing licensing examination in order to investigate its internal structure using DETECT. The DETECT program provided the number of clusters and which item belonged to which cluster. Table 3 shows that the 3 clusters for each sub-domain were suggested, except for psychiatric nursing and medical health legislation. The number of items assigned to each dimension was equally distributed in the Korean nursing licensing examination, except that 1 item from the subject of psychiatric nursing was assigned into a single cluster. This process provides information regarding the exploratory internal structure of the Korean nursing licensing examination. Since the Dp* of community health nursing content was greater than 1.0, it contained sizable multidimensionality. All *r* values were greater than 0.8, meaning that each subject could approximately be regarded as a simple multidimensional structure. In addition, confirmatory information was obtained of the theoretically suggested internal structure of the Korean nursing licensing examination. The results of the DETECT program can be compared with the theoretically expected content specification to examine whether it correctly detected the number of sub-domains and properly assigned items for each dimension.

## Discussion

Specific patterns were observed in the performance of DETECT in the simulation study. Overall, the DETECT procedure tended to overestimate the number of dimensions in the simulation study. In more detail, the DETECT procedure tended to determine a higher number of dimensions than the true number of dimensions when the guessing parameter was included. However, when the sample size was large and the correlation levels among dimensions were lower, the DETECT procedure tended to determine the number of dimensions well. In the real data study, the DETECT program performed well in detecting the number of dimensions and assigning items into each dimension. This result implies that the DETECT method can be a useful tool for researchers to investigate the internal structure of a complex assessment, such as a licensing/certification examination that possesses relatively many domains and content areas.

The major limitation of this study is that the simulation data were generated based on multidimensional IRT with specific multidimensional conditions. Therefore, a variety of further conditions, such as more dimensions and different associations among dimensions, should be investigated in future studies. Furthermore, since this study generated data based on a compensatory multidimensional IRT model, the performance of the DETECT program should be examined for non-compensatory multidimensional IRT in the future.

Most current licensing/certification examinations possess more than 4 sub-domains. Evidence of their internal structure is an important source for validation studies of these examinations. The internal structure provides the number of domains measured by an assessment and the relationships between domains and items. Multidimensionality does not mean low validity. If a test is detected to have multidimensionality, then the number of clusters should be compared to the content objectives and we can apply a multidimensional IRT or diagnostic classification model with that information. Appropriate information regarding the internal structure of an assessment enables the proper inference, interpretation, and application of examination scores. The DETECT program is a useful tool for investigating the internal structure of a complex assessment. Moreover, recently, the cognitive diagnostic model has frequently been applied to analyze complex examinations containing several sub-domains. In such cases, the DETECT program can be used as a preliminary analytical tool to investigate the number of sub-domains and the item alignment for each sub-domain. Therefore, if the purpose of a study is to investigate the internal structure of an examination that contains many sub-domains, the DETECT program would be a suitable method to obtain useful information. This study may be useful for practitioners who are considering the investigation of the internal structure of licensing/certification examinations as a preliminary study.

## Figures and Tables

**Table 1.** Content specification of the Korean nursing licensing examination

Subjects	Items
Adult health nursing	80
Maternity nursing	40
Pediatric nursing	40
Community health nursing	40
Psychiatric nursing	40
Nursing management	40
Fundamental nursing	30
Medical health legislation	20
Total	330

**Table 2.** Average detection rate of dimensions with 100 replications under several conditions

No. of dimensions	Correlation among dimensions	Sample size	Guessing parameter	Average no. of clusters	Average value of Dp^*^
2	0.3	100	c= 0	2.2	0.294
			c = 0.25	3.3	0.362
		1,000	c= 0	3.1	0.368
			c = 0.25	3.4	0.389
	0.7	100	c= 0	2.1	0.284
			c = 0.25	2.2	0.322
		1,000	c= 0	2.1	0.372
			c = 0.25	3.1	0.274
4	0.3	100	c= 0	5.3	0.311
			c = 0.25	6.1	0.382
		1,000	c= 0	4.2	0.374
			c = 0.25	5.4	0.399
	0.7	100	c= 0	3.8	0.284
			c = 0.25	4.1	0.352
		1,000	c= 0	3.6	0.255
			c = 0.25	5.1	0.266

**Table 3.** Maximum DETECT values of each content area for the Korean nursing licensing examination

Subjects	No. of clusters (no. of items in each cluster)	Dp^*^	r
Adult health nursing	3 (C1 = 21, C2 = 31, C3 = 28)	0.626	0.909
Maternity nursing	3 (C1 = 11, C2 = 16, C3 = 13)	0.634	0.869
Pediatric nursing	3 (C1 = 14, C2 = 12, C3 = 14)	0.623	0.931
Community health nursing	3 (C1 = 13, C2 = 15, C3 = 12)	1.503	0.864
Psychiatric nursing	4 (C1 = 13, C2 = 14, C3 = 12, C4 = 1)	0.379	0.890
Nursing management	3 (C1 = 14, C2 = 14, C3 = 12)	0.258	0.926
Fundamental nursing	3 (C1 = 10, C2 = 10, C3 = 10)	0.631	0.933
Medical health legislation	4 (C1 = 6, C2 = 3, C3 = 3, C4 = 8)	0.228	0.921

C1, cluster 1; C2, cluster 2; C3, cluster 3; C4, cluster 4.

## Supplementary materials

Supplement 1. Data files are available from https://doi.org/10.7910/DVN/O3NUPJ. Item order of data was transformed randomly in each subject.

Supplement 2. Audio recording of the abstract.
